# Draft Genome Sequences of Bacillus subtilis Strains TNC1(2019), TNC3(2019), and TNW1(2019), as Well as Bacillus velezensis Strains TNC2(2019) and TNW2(2019), Isolated from Cabbage Kimchee

**DOI:** 10.1128/MRA.00085-20

**Published:** 2020-06-04

**Authors:** Gabriel G. Perron, Tejaswee Neupane, Rachael A. Mendoza

**Affiliations:** a Department of Biology, Reem-Kayden Center for Science and Computation, Bard College, Annandale-on-Hudson, New York, USA; b Bard Food Lab, Center for Experimental Humanities, Bard College, Annandale-on-Hudson, New York, USA; c Center for the Study of Land, Air, and Water, Bard College, Annandale-on-Hudson, New York, USA; University of Maryland School of Medicine

## Abstract

We report the draft genome sequences of five novel *Bacillus* strains isolated from five different batches of fermented Napa cabbage kimchee. Strains TNC1(2019), TNC3(2019), and TNW1(2019) were identified as Bacillus subtilis, while TNC2(2019) and TNW2(2019) were identified as Bacillus velezensis.

## ANNOUNCEMENT

*Bacillus* is a genetically and metabolically diverse genus of bacteria that have been used for the development of environmental and medicinal tools (1). The genus also plays an important role in the making of many traditional fermented foods, such as natto and miso, two products made from fermented soybeans (2, 3). For this reason, the characterization of novel *Bacillus* strains is of interest for the development of novel agricultural and biotechnological applications such as probiotics (4).

To investigate the genetic diversity of *Bacillus* strains in fermented food, we isolated the most frequent bacterial strain found in five different batches of Napa cabbage kimchee. In addition to Napa cabbage, the five kimchee preparations included the same quantities of Asian pear, garlic, chili powder, dried shrimp, and ginger and were fermented for 14 days. For each ferment, bacterial count and diversity were estimated from a 10-fold serial dilution initiated with 1 ml of brine. We then plated 100 μl of each dilution onto LB plates and incubated the plates at 37°C for 48 h. For each sample, we picked one colony from the most frequent colony type, which was then grown in LB broth at 37°C for 48 h. The bacterial cultures were then streaked onto LB plates to confirm the unique colony morphology, and we used 1.8 ml of overnight cultures to extract genomic DNA using the DNeasy UltraClean microbial kit (Qiagen, Hilden, Germany). Whole-genome sequencing was conducted at the Microbial Genome Sequencing Center, LLC (Pittsburgh, PA), using the 151-bp paired-end read libraries designed for the Illumina MiSeq platform. We obtained a total of 3,653,401 raw reads for TNC1(2019), 3,551,954 raw reads for TNC2(2019), 3,581,451 raw reads for TNC3(2019), 3,863,404 raw reads for TNW1(2019), and 3,466,478 raw reads for TNW2(2019).

We assembled the genomes using the bioinformatic pipeline described by Shrestha et al. (5), using default parameters unless specified otherwise. Briefly, raw reads were trimmed using Trimmomatic v0.36 with the following parameters: slidingwindow, 4:15; leading, 3; trailing, 3; and minlen, 50 (6). Trimmed reads were then assembled using the SPAdes *de novo* assembler v3.11, testing assemblies with kmer values of 21, 33, 55, 77, 99, and 127 (7); contigs smaller than 500 bp or with low coverage (lower than or equal to 2×) were filtered out. Assembly statistics were estimated using QUAST v4.5 (8) and BBMap v35.82 (9). We also estimated genome completeness and confirmed that no contamination was detected using CheckM v1.0.18 (10). Finally, species identity, based on DNA-DNA hybridization (DDH) values calculated with the closest relative, was estimated for each strain using the genome BLAST distance phylogeny approach (11), as implemented on the Type (Strain) Genome Server (TYGS) using default settings (12).

We found that the draft genomes of strains TNC1(2019), TNC3(2019), and TNW1(2019) were most closely related to Bacillus subtilis subsp. *subtilis* (ATCC 6051) (NCBI accession number ASM608879v1), with DDH values of 91.7%, 90.0%, and 86.8%, respectively. The three draft genomes ranged in size from 4,044,726 bp to 4,080,193 bp, with an average G+C content of 43.6% and median coverage of 106.7×. We found that the draft genome of strain TNC2(2019) was most closely related to Bacillus velezensis NRRL B-41580 (NCBI accession number ASM146182v1), a type strain isolated from river sediments in Spain (13), and that of strain TNW2(2019) was most closely related to Bacillus velezensis FZB42 (NCBI accession number ASM1578v2), a strain formerly known as Bacillus amyloliquefaciens subsp. *plantarum* FZB42 and isolated from beet rhizosphere (14). The two strains had an average G+C content of 46.3%, median coverage of 100×, and DDH values of 93.1% and 91.0%, respectively.

### Data availability.

The draft genomes and raw reads for each strain have been deposited in DDBJ/EMBL/GenBank under the accession numbers listed in Table 1.

**TABLE 1:** Summary of the draft genome sequences for the *Bacillus* sp. strains described in this study

Isolate^*a*^	Coordinates	Closest species match (DDH [%])	No. of contigs	Genome size (bp)	Completeness (%)	G+C content (%)	*N*_50_ (bp)	Median read depth (×)	Genome accession no.	SRA accession no.
TNC1(2019)	42°1′12ʺN, 73°54′28ʺW	*B. subtilis* ATCC 6051 (91.7)	15	4,080,193	99.59	43.7	2,103,272	105	JAAEBK000000000	SRR10969390
TNC2(2019)	42°1′49ʺN, 73°54′17ʺW	*B. velezensis* NRRL B-41580 (93.1)	24	4,032,556	99.59	46.4	414,810	101	JAAEBJ000000000	SRR10969389
TNC3(2019)	42°1′47ʺN, 73°54′16ʺW	*B. subtilis* ATCC 6051 (87.7)	18	4,070,250	99.59	43.7	1,024,243	102	JAAEBI000000000	SRR10969388
TNW1(2019)	42°1′12ʺN, 73°54′28ʺW	*B. subtilis* ATCC 6051 (86.1)	23	4,044,726	99.59	43.5	1,057,433	113	JAAEBH000000000	SRR10969387
TNW2(2019)	42°1′26ʺN, 73°54′23ʺW	*B. velezensis* FZB42 (91.0)	40	4,050,273	99.59	46.1	295,280	98	JAAEBG000000000	SRR10969386

a All isolates were obtained from kimchee in Annandale, New York.
